# Effects of nurse telesupport on transition between specialized and primary care in diabetic patients: study protocol for a randomized controlled trial

**DOI:** 10.1186/s13063-017-1954-z

**Published:** 2017-05-18

**Authors:** Ana Marina Moreira, Roberta Marobin, Dimitris Varvaki Rados, Camila Bergonsi de Farias, Sabrina Coelli, Bárbara Luiza Bernardi, Lívia de Almeida Faller, Laura Ferraz dos Santos, Ana Maria Matzenbacher, Natan Katz, Erno Harzheim, Sandra Pinho Silveiro, TelessaudeRS

**Affiliations:** 1 Post Graduate Program in Medical Sciences – Division of Endocrinology, Federal University of Rio Grande do Sul (UFRGS), Porto Alegre, Brazil; 2 Division of Epidemiology, Federal University of Rio Grande do Sul, Porto Alegre, Brazil; 3 Serviço de Endocrinologia do Hospital de Clínicas de Porto Alegre, Rua Ramiro Barcelos, 2350, 4 andar, Porto Alegre, RS CEP 90035-903 Brazil

**Keywords:** Type 2 diabetes mellitus, Phone calls, Telemedicine, Glycemic control

## Abstract

**Background:**

According to the Global Diabetes Plan, a unified health system with preventive and educational strategies is essential to proper diabetes care and primary settings should be the main site of care. In Brazil, there is limited access to outpatient hospital diabetes services, while primary-care diabetes support is underutilized. Telemedicine can be a useful adjunct to support discharge of stable patients with type 2 diabetes to the primary care setting. In this paper, we present a randomized controlled trial (RCT) protocol designed to evaluate the effects of telehealth support for stable type 2 diabetes patients discharged from hospital outpatient diabetes clinics.

**Methods:**

We designed a RCT. Patients with stable type 2 diabetes (glycated hemoglobin < 8%) considered eligible for discharge from specialized to primary care will be included. Those with uncontrolled ischemic heart disease, severe neuropathy, and stage IV/V nephropathy will be excluded. Enrolled patients will be randomized into two groups: follow-up supported by periodic phone calls by a nurse (intervention group) plus primary care or routine primary care only (control group). The intervention group will receive regular telephone calls (every three months for one year) and will have a toll-free number to call in case of questions about disease management. The main outcome measure is a comparison of glycemic control between groups (assessed by glycated hemoglobin) at one-year follow-up.

**Discussion:**

We plan to evaluate the effectiveness of a telephone-based intervention on glycemic control in patients with type 2 diabetes followed by primary care teams. Telemedicine can be an important adjunct in type 2 diabetes management, improving patient education and knowledge about the disease. Furthermore, it can help the healthcare system by alleviating overload in specialized care settings and supporting the stewardship role of primary care.

**Trial registration:**

Clinical Trials, NCT02768480. Registered on 29 April 2016.

**Electronic supplementary material:**

The online version of this article (doi:10.1186/s13063-017-1954-z) contains supplementary material, which is available to authorized users.

## Background

According to the International Diabetes Federation (IDF), in 2015, the global prevalence of diabetes was 415 million adults. Over 14.3 million of these adults live in Brazil, making it the country with the fourth largest number of persons living with diabetes [1]. Due to this large population of patients, mostly affected by type 2 diabetes, no health system can be planned without coordinating between primary and specialized care. The Global Diabetes Plan suggests that diabetes management in primary care could be key to achieving glycemic control and preventing chronic complications [2].

Brazil has experienced major changes in how healthcare is provided in the last 20 years: a structured and coordinated health system was created and primary care was defined as the main setting of healthcare. Despite these changes, the population has limited access to primary care, which has little problem-solving capacity, and specialized clinics are overcrowded.

Since 2007, TelessaúdeRS – UFRGS, a multiple-intervention telemedicine project, has been supporting primary care providers to improve management of chronic diseases. Our experience shows that many unnecessary referrals can be avoided through teleconsultations and training of primary care teams [3, 4]. We plan to explore whether telehealth strategies can have similar positive effects on patients being discharged from specialized to primary care. For the present project, we specifically aim to assess the effects of a simple remote monitoring strategy for the transition of type 2 diabetes care from specialized to primary facilities. This report follows the guidelines provided in the SPIRIT Statement [5]. We presented a standardized checklist with recommended items of SPIRIT Statement (Additional file 1).

## Methods

### Study design and settings

This open-label randomized controlled trial will seek to evaluate the effects of patient-directed phone calls as a means of supporting the care of individuals discharged from an outpatient hospital-based tertiary care diabetes clinic to primary care. The study will be performed at an outpatient diabetes clinic at Hospital de Clinicas de Porto Alegre, an academic hospital in southern Brazil. This hospital is a TelessaúdeRS partner in offering support to primary care.

### Eligibility criteria

#### Inclusion criteria

Patients eligible for the trial must comply with all of the following criteria at randomization: established diagnosis of type 2 diabetes; and HbA1c level < 8% (HPLC method).

All patients that meet the inclusion criteria above are able to enter in the randomization, regardless of the treatment in use to manage diabetes (diet, oral antihyperglycemic medication, or insulin). The research team will identify patients meeting the inclusion criteria.

#### Exclusion criteria

Patients with the following criteria will not be included in the study: stage IV or V nephropathy; uncontrolled ischemic heart disease; or severe autonomous or peripheral neuropathy.

### Intervention

**Fig. 1:**
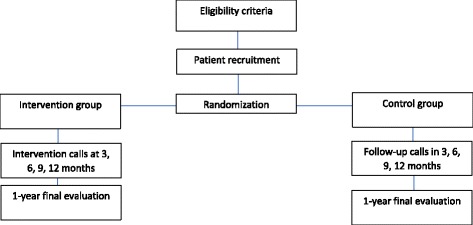
Study *flowchart*: describes randomization and follow-up of participants

**Fig. 2:**
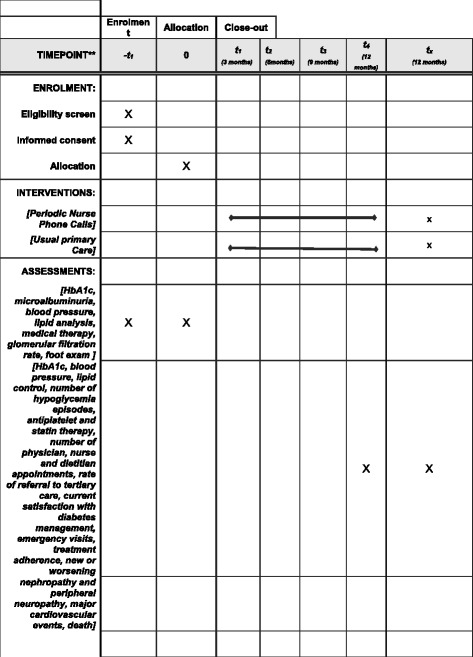
SPIRIT figure: Summarizes the allocation, interventions, and outcomes of the study

Study flowchart, procedures and evaluations are depicted in Figs. 1 and 2. All patients will receive an illustrated brochure with information about diabetes care and a standardized discharge document. The discharge note will be directed to the primary care team and will present the patient’s medical history, current type 2 diabetes care regimen, individualized glycemic target, and management plan. The TelessaúdeRS toll-free number will also be mentioned in the discharge note as a tool for helping primary care manage the patient.

Patients randomized to the intervention group will receive a toll-free number to call nurses who can review their treatment and address any questions about their diabetes care. In addition, they will receive quarterly calls from the nurse team to review topics on diabetes education and non-pharmacologic and pharmacologic treatment. In the phone calls, nurses will do educational interventions, reviewing therapy adherence, side effects, techniques of insulin application, diet adherence, foot care, frequency of physician visits, and will give advices about hypoglycemia. Due to national legal restrictions, no treatment adjustments will be performed; therefore, if medication adjustments are needed, patients will be oriented to consult with the primary care provider.

Patients randomized to the control group will be followed exclusively by the primary care team. These patients will also receive follow-up phone calls at the same times as the intervention group (at three, six, nine, and 12 months), but without any diabetes intervention. If necessary, in both groups, treatment adjustments will be performed by the primary care physician, regardless of telemedicine actions.

### Outcomes

One year after enrollment, both groups will undergo a standardized evaluation of current diabetes management, health services support, and treatment adherence. All participants will also undergo blood and urine collection for laboratory assessment.

The primary outcome will be glycemic control measured by HbA1c. The following outcomes will also be evaluated: blood pressure control evaluation (after 5 min of seated rest); lipid control; number of severe hypoglycemia episodes; antiplatelet and statin therapy; number of physician, nurse, and dietitian appointments; rate of referral to tertiary care; current satisfaction with diabetes management; emergency visits because of acute complications of diabetes; treatment adherence; new or worsening nephropathy and peripheral neuropathy; major cardiovascular events; and death.

### Sample size

We calculated a sample of 63 participants in each treatment arm to identify a between-group difference of 1% in HbA1c after one year, considering a standard deviation of 2%, an alpha of 0.05, and a statistical power of 80%.

### Recruitment

Study monitors will be responsible for identifying eligible patients at the time of discharge. After discharge, the research team will explain the study procedures and will invite eligible patients to join the study. After providing written informed consent, the patients will be randomized to the intervention (primary care with nurse-led telephone support) or control (primary care alone) groups. Patients will complete a standardized questionnaire and undergo clinical and laboratory assessment.

### Randomization and participant allocation

The random number sequence will be generated online (via randomization.com), using a 1:1 ratio, with blocks of four and six patients. The researcher responsible for randomization will not be involved in patient recruitment or enrollment. No stratification method will be used. Patients will be allocated sequentially, using dark-brown sealed envelopes, which will be opened only after patients have provided consent and the first assessment has been performed.

### Blinding

The study is not blinded for trial participants, care providers, or outcome assessors. Statistical analysis will be performed in blind fashion.

### Data collection methods

Study personnel will collect baseline data with a standardized questionnaire. Blood pressure will be measured after a 5-min rest. Information about foot sensitivity, ophthalmologic evaluation, glycemic control (HbA1c, fasting glucose), renal function (creatinine, glomerular filtration rate [GFR], and albuminuria), and lipid profile will be obtained from medical records. Follow-up information will be collected with a standardized questionnaire by telephone quarterly to assess patient’s participation and minimize follow-up dropouts. After the completion of the study, the final assessment will be performed with a standardized questionnaire. The questionnaires will evaluate the following: demographic, ethnical, and education level data; drug or alcohol use; treatment of diabetes; cardiovascular history; associated co-morbidities; hospital admissions; and smoking status. Furthermore, the final questionnaire will evaluate the treatment satisfaction, emergency visits for a diabetes reason, rate of hypoglycemia, number of primary care visits to review diabetes, and rate of referrals to tertiary care. The Brief Medication Questionnaire will be used to assess adherence and comprehension of therapy on baseline and follow-up. Blood pressure, body weight, and height and foot exam will be performed and blood and urinary samples will be collected.

### Statistical methods

Variables with normal distribution will be presented as means ± standard deviation; asymmetric variables will be presented as median and interquartile range. Categorical variables will be presented as total counts and percentages. Normal continuous variables will be compared with Student’s t-test and categorical variables will be compared with the chi-square test. No interim analysis is planned. Main outcomes will be presented both with intention-to-treat (all randomized patients) and per-protocol (patients who received at least one telephone contact) analyses. We will use Cox regression for outcome analysis.

### Data monitoring and auditing

Due to the lack of expected harms and the nature of the intervention and outcomes, no data monitoring committee will be created. The study coordinator will perform constant auditing of data and study conduct, but no external auditing process is planned.

### Harms

There are no expected harms to study participants, except risk of data violation. The investigators will mitigate this risk by codifying participants with an identification number. Although the intervention group is expected to be more adherent to treatment, systematic differences in diabetes management are not expected.

## Discussion

Diabetes mellitus is a chronic condition associated with high mortality, reduced quality of life, and increased health cost. In particular, patients living in developing countries experience additional problems, such as poor support and limited access to healthcare services [6]. In this context, an integrated, multidisciplinary health system focused on prevention and education strategies is the key to provide adequate care to diabetic patients. Such integrated systems should focus on providing highly effective primary care, which should be able to treat the majority of diabetic patients, and refer only those with severe complications and refractory disease. Furthermore, clinical support through telemedicine interventions could strengthen primary care and enhance its quality [7].

Brazil has an excessive number of referrals and large waiting lists for referral to specialized care, with waiting times of months to years. Moreover, specialized services are unable to meet the demand for care, which leads to overloading of endocrinology clinics with unnecessary visits.

Our study aims to explore if we can improve the performance of primary care by supporting patients in the transition from specialized to primary care through the use of telehealth services. We aim to include well-controlled patients (defined as HbA1c < 8%) who no longer benefit from specialized care to glycemic treatment adjustment. We expect to answer whether a periodic, systematic intervention focused on education and adherence can improve diabetes control and whether this strategy can support management, maintaining, or improving the treatment offered in primary care. We will explore an innovative strategy to optimize the transition of care, based on a tool that has proven benefit in diabetes management. As future perspectives, if this intervention shows benefits to diabetic patients being discharged from specialized care, an economic assessment is planned to evaluate the costs of the intervention and to discuss with stakeholders the widespread implementation.

In one previous study of low-income urban adults, periodic phone calls had a superior effect on glycemic control compared to printed education material (control). After one year, a significant decrement in HbA1c was demonstrated in the intervention group (–0.23% ± 0.11% versus +0.13% ± 0.13%; *p* = 0.04), with a better effect in patients who received more calls [8]. Similar results were found in a larger sample with fewer phone calls [9]. As expected, patients with the worst baseline glycemic control had greater reductions in HbA1c.

On the other hand, a recent meta-analysis of five studies that compared phone-call intervention with usual care in patients with type 2 diabetes showed a non-significant decrement of HbA1c values in the intervention group (pooled mean difference, –0.38%; 95% confidence interval, –0.91 to 0.16) [10]. Thus, the role of periodic educational phone calls on glycemic control remains unclear, despite some evidence for improvement [11].

Despite such evidence of improved glycemic control, long-term studies are lacking. The current literature has no information about the effects of such interventions on chronic complications of diabetes, cardiovascular events, or mortality, and the majority of studies have employed follow-up periods between six months and one year. A future long-term evaluation of the aforementioned outcomes is important to analyze the effectiveness of this intervention and decide if it is indeed useful to improve primary care, considering the cost benefit.

In summary, we believe that telehealth can be a useful tool for diabetes management, facilitating patient self-management and the transition from specialized to primary care. The results of this study will help us define whether telephone nurse support should be made widely available as a tool to reduce overcrowding of specialized services.

### Trial status

This study is currently recruiting participants. The recruitment started at June 2015 and is expected to end at June 2017.

## Additional file

Additional file 1: SPIRIT Checklist: A standard checklist required for publication of study protocols. (DOC 122 kb)

## Abbreviations

GFR: Glomerular filtration rate
HbA1c: Glycated hemoglobin
UFRGS: Universidade Federal do Rio Grande do Sul
